# The Hygiene of Food

**Published:** 1881-07

**Authors:** 


### THE HYGIENE OF FOOD.

The breakfast is probably the most important meal of the day. Mr. Ernest Harr writes in reference to it in the *Sanitary Record*, London, England, as follows :

The skilled cooking of economic materials affords a theme on which it is popularly supposed to be easy to preach, and far from easy to carry doctrine into practice. I shall speak only of the mode of preparation of the very simplest and cheapest kinds of food in the simplest and cheapest way. I shall begin with breakfast, and breakfast foods. I should like to see the tea-pot abolished from the breakfast table. I believe tea to be a drink utterly unsuited for an early morning meal, and one which has only come into general use because it is the easiest sort of hot infusion which bad cooks, careless house-wives and thoughtless mothers can prepare. Breakfast should be digestible, warm, abundant, unexciting, nourishing. I am not going to quarrel with bread and butter, especially other people's. It is a typically good food, though not always presented in the most agreeable form, and far from being the most appetizing or cheapest of its class. Bread and butter, and hot cocoa, make a very good breakfast for working people, but not perhaps the cheapest they can get, or the handiest. I believe very firmly in our good English household white bread. One hears a good deal, and reads a good deal, of the waste in grinding off the outside husk, which contains nutritious gluten. The apparent economic waste is palpable enough. On the other hand, the silicated husks of all cereals is apt to be irritating. It hurries the digestion, quickens the passage of food through the intestinal tract, and I am inclined to believe that the actual physiological waste is greater in a brown bread than in a white bread diet. It is easy to take a superficial view of this question, and superficial reformers are always wanting to turn the world upside down. The instinct which has led to the preference of white bread over brown, in places where the two can be had side by side, is nothing else than the crystallized experience which has taught people unconsciously that they are more comfortable after eating the white bread, and that the solid household bread, which is the staple food of the working classes of this country, is in the end most sustaining. A good deal is to be said in favor of some of the forms of "whole meal bread," in which the husk is partially ground off, and the inner pellicle of the grain is very finely ground, and mixed in that condition with the white flour. Moreover, it is undoubtedly a fact that under certain circumstances, in lymphatic temperaments, and in conditions tending towards scrofula, where the diet has to be carefully supervised, and in certain forms of dyspepsia, where something like mechanical excitation of the intestinal tract is useful, whole meal bread is an extremely valuable article of diet.

But those are cases which I am not considering. For the working man, for the poor man, and for every day use, I doubt whether anything has yet been produced in any country of the world which is equal to the English household bread. But when we pass out of this category, and come to consider what is to be the cheap, warm, nourishing breakfast with which, at the least trouble and smallest expense, and with the greatest success, we can nourish our children, ourselves, our servants and our laborers, then we have to consider the claims of an immense class of cereals, of which, to our shame, we make but little use in this country, and thereby suffer a great economic and hygienic loss. Wheat is a costly cereal, and it is not the most nourishing, nor does it lend itself well to those pleasant, wholesome, nutritious and comforting forms of food known as porridges, which do form the staple breakfast throughout Scotland and throughout the vast American continent, which is now peopled with English, Scotch and Irishmen, and from which we have so much to learn and so much to gain. * * * * Hominy porridge is the staple breakfast of the American continent. For young people, for reasonably quiet people, for dyspeptics, for working people, for bankers and brokers, who want to keep their digestion in good order, and to be able to work satisfactorily, hominy porridge is the only food ; hominy is nothing else than a fine kind of Indian corn, ground roughly and largely like Scotch oatmeal, and the way to make the porridge is to soak it in cold water all night, and to boil it for half an hour in the morning, stirring it frequently to prevent it from burning. When boiled, each grain should be soft and separate, like well boiled rice. It should retain its opaque whiteness, and should not be watery or semi-transparent, or else it may be known to be over-cooked. Neither should it run into masses, or coagulate in lumps ; all these are the indicia of bad cooking. They may be easily avoided, and hominy which does not come up to table with every grain soft and separate, and showing a pure, opaque, pearl-like whiteness, should be sent down again and devoted to some other use, such as frying in slices. When served in a hot bowl (with a pile of hot plates), it is best eaten with milk and sugar by the luxurious. To children and simple minded people it is delicious with skim-milk and treacle, and of all the cheap, wholesome, digestible, delicious breakfasts which the world affords, I do not know any which can compare with a dish of hot hominy with skim-milk and golden syrup. It would delight the heart of any British child. There is no epicure who retains a palate capable of appreciating simple purity of flavor, or a mind capable of appreciating the best gifts of nature prepared for gastronomic enjoyment, who will not find in such a dish of hominy one of the most perfect luxuries which could be put before him. I should like to hear that some of the royal children take hominy, skim-milk and golden syrup every morning for breakfast. It would make it fashionable, and such a dish requires only to be fashionable in this country in order to become universal, and to be as popular in the palace as in the peasant's cottage. One practical inconvenience is sometimes found in preparing porridge, and that is the necessity of steeping the hominy over night, and spending half an hour in boiling it in the morning.

